# Mesenchymal stromal cells to modulate immune reconstitution early post-hematopoietic cell transplantation

**DOI:** 10.1186/s12865-015-0135-7

**Published:** 2015-12-16

**Authors:** Elizabeth O. Stenger, Lakshmanan Krishnamurti, Jacques Galipeau

**Affiliations:** Aflac Cancer and Blood Disorders Center, Children’s Healthcare of Atlanta, Emory University, 1405 Clifton Road, Atlanta, GA 30322 USA; Department of Hematology and Medical Oncology, Winship Cancer Institute, Emory University, 1365 Clifton Road, Atlanta, GA 30322 USA

## Abstract

Mesenchymal stromal cells (MSCs) are multipotent progenitor cells known to modulate the immune system and to promote hematopoiesis. These dual effects make MSCs attractive for use as cellular therapy in hematopoietic cell transplantation (HCT). MSCs can be used peri-HCT or pre-engraftment to modulate immune reconstitution, promoting hematopoietic stem cell (HSC) engraftment and/or preventing graft-versus-host disease (GVHD). Pre-clinical studies have demonstrated that MSCs can potentiate HSC engraftment and prevent GVHD in a variety of animal models. Clinical trials have been small and largely non-randomized but have established safety and early evidence of efficacy, supporting the need for larger randomized trials.

## Background

### Mesenchymal Stromal Cells in Hematopoietic Cell Transplantation

Mesenchymal stromal cells (MSCs) are multipotent progenitors that promote hematopoiesis and have unique immunoregulatory properties, making them attractive for use as cell-based therapy during and post hematopoietic cell transplantation (HCT). They co-localize with hematopoietic stem cells (HSCs) in the normal bone marrow (BM) niche, producing factors that promote and recruit HSCs and regulate their function [1]. MSCs also regulate both innate and adaptive immune responses through effects on various immune cells, particularly T cells and antigen-presenting cells [2–4]. Specifically, they down-regulate immune responses by promoting regulatory T cells and inhibiting cytotoxic T cell proliferation. Human MSCs have been shown to function via IFN-γ-dependent upregulation of indoleamine 2,3-dioxygenase (IDO), whereas inducible nitric oxide species are important for the function of MSCs from mice and other species [5, 6].

**Fig. 1:**
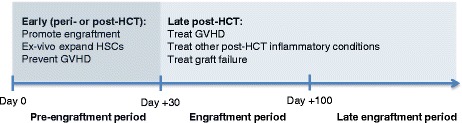
Early (peri- or post-transplant) versus late administration of MSCs in HCT. MSCs can be administered to HCT patients in either early or late time periods. The early time period constitutes either peri- or early post-transplant, which includes the use of MSCs to promote engraftment or to prevent GVHD. MSCs can potentiate engraftment via direct infusion peri-transplant or via ex-vivo co-culture with HSCs. The late time period constitutes the late post-engraftment period where MSCs can be infused to treat GVHD or other inflammatory conditions (such as hemorrhagic cystitis) or to treat graft failure

The surge in pre-clinical and clinical studies of MSCs led the International Society for Cellular Therapy to define minimal phenotypic and functional criteria for MSCs in 2006 [7, 8]. Numerous clinical trials have demonstrated the feasibility of *ex vivo* expansion of MSCs and the safety of MSC infusion. There are currently more than two-hundred and fifty open MSC trials (https://www.clinicaltrials.gov/). In HCT, the use of MSCs can be broadly categorized into early (peri-transplant or early post-transplant) versus late administration (late post-transplant; Fig. 1). MSCs have been given in the late post-transplant period primarily as treatment for graft-versus-host disease (GVHD), and there are currently over twenty open studies for this indication registered at https://www.clinicaltrials.gov/. The use of MSCs to treat GVHD has been reviewed extensively elsewhere [9, 10]. Studies to date show promise in certain subsets of patients, specifically in pediatric rather than adult patients and in the treatment of liver and gut rather than skin GVHD. This review will focus exclusively on the early administration of MSCs in HCT to modulate immune reconstitution and engraftment.

### Early Administration of MSCs in HCT

**Fig. 2:**
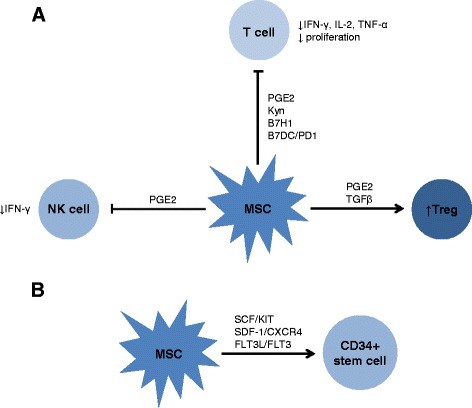
Potential mechanisms of MSC potentiation of engraftment. Pre-clinical studies (in vivo small animal and in vitro with human MSCs) suggest that MSCs function via interaction with other immune cells (Fig. 2 **a**) and with donor CD34^+^ hematopoietic stem cells (HSCs; Fig. 2 **b**). MSCs may inhibit activated residual recipient immune cells, in particular T lymphocytes and natural killer cells which are known to be drivers of HCT rejection, and/or may promote other regulatory immune cell populations, such as regulatory T cells. As shown in Fig. 2a, some potential mechanisms of this former effect are demonstrated, including cell-cell interaction (such as through B7H1 or B7DC/PD1 on MSCs) or secretion of small molecules (such as PGE-2 through COX2, kynurenine through IDO, and IL-10). MSC likely interact with HSCs through cell-cell interactions, which more likely occur before HSCs reach the bone marrow niche and may lead to HSCs being directed to the niche and/or to increased HSC survival (Fig. 2b)

Based upon their dual role in supporting hematopoiesis and modulating immunity, MSCs have been studied in the peri- and early post-transplant period to promote engraftment and immune reconstitution. Given known MSC immunomodulatory capacity and pre-clinic studies of MSCs given peri-HCT, MSCs likely promote engraftment through the inhibition of recipient immune cells that remain following transplant conditioning and through the promotion of other immunomodulatory cell populations, such as regulatory T cells (Fig. 2a) [3, 5, 11, 12]. As T lymphocytes and natural killer cells are a primary driver of graft rejection, it is likely that MSCs impact on these cell populations, both through cell-cell interactions and through secretion of soluble factors [3, 5, 11, 12]. Additionally, it is probable that MSCs also enhance engraftment through interaction with donor CD34^+^ hematopoietic stem cells (HSCs), potentially by directing HSCs to the bone marrow niche or increasing their survival (Fig. 2b) [13–16]. This MSC/stem cell interaction may occur either before stem cells reach the bone marrow niche or in the niche itself, although the majority of clinical studies fail to show engraftment of infused MSCs, making it more likely to occur outside of the marrow.

Engraftment of HSCs is a considerable barrier to successful transplant, particularly in HCT for non-malignant diseases (NMD). Graft failure is predominantly immune-mediated, wherein recipient T cells play a dominant role in rejecting donor hematopoietic cells [17, 18]. The risk of graft failure therefore increases with the degree of human leukocyte antigen (HLA) mismatch, intensity of pre-transplant conditioning, and ex-vivo graft T cell depletion (TCD) [17]. Engraftment following umbilical cord blood transplantation (CBT) has been particularly challenging in patients with NMD, which can be partly, but not completely overcome with increasing cell dose [19, 20].

#### MSCs to Promote Engraftment in Experimental HCT Models

MSCs given in the peri-transplant period have been shown to promote engraftment across a variety of pre-clinical models and using different MSC sources (Table 1). The majority of murine studies have utilized a NOD/SCID model giving sub-lethal doses of radiation prior to transplantation. MSC isolated from random donor human fetal lung [21, 22], fetal BM [22], adult BM [22], and placenta [13] enhance engraftment of a single human CD34^+^ CB, although results were not statistically significant using placental MSCs. Both placental [13] and BM [23] MSCs enhance double CB engraftment in the NOD/SCID mouse model. Third-party human BM MSCs also increase the engraftment of a double CB compared to single CB with MSC co-transplantation (including with 5 locus mismatch) and to double CB alone [23]. Both human placental and BM MSCs also significantly decrease single cord dominance in this model [13, 23].

**Table 1:** Use of MSCs to promote engraftment in murine models

HSC source	Model	Conditioning	MSC source	MSC dose	Outcome	Ref
Human UCB CD34^+^cells	NOD/SCID mice	3.5 Gy	Human fetal lung	1 × 10^6^	3-4 fold increased donor CD45^+^ (lymphoid & myeloid, not B cell)	21
Human UCB CD34^+^cells	NOD/SCID mice	3.5 Gy	Fetal lung, liver, or BM or adult BM	1 × 10^6^	Fetal lung & BM & adult BM MSCs increased engraftment of donor CD45^+^ in BM, PB, or spleen	22
Human UCB CD34^+^cells (single vs double)	NOD/SCID mice	3.25 Gy	Human placenta	4 × 10^4^	Increased engraftment (total & CD34^+^), no effect on CD19^+^ Decreased single cord dominance	13
Human UCB CD34^+^cells (single vs double)	NOD/SCID mice	3.5 Gy	Human BM	NR	Increased engraftment, decreased single cord dominance	23
Human PB CD133^+^cells	NOD/SCID mice	3 Gy	Human BM; CD271^+^ & PA-MSC	1 × 10^5^(1:1; HSC:MSC) 7 × 10^5^ (1:8)	Increased engraftment (CD45^+^, CD33^+^, CD19^+^), no effect on CD41a^+^, CD3^+^, CD56^+^ Increased engraftment (CD271^+^ > PA-MSC), comparable increase CD33^+^ & CD41a^+^, CD271^+^ increase lymphoid & decrease NK cells	24
Human UCB CD34^+^cells	NOD/SCID mice	3.5 Gy	Human BM (STRO-1^+^ or STRO-1^−^)	1 × 10^6^	STRO-1^−^ MSC increased engraftment of donor CD45^+^ in BM, PB, or spleen more than STRO-1^+^, STRO-1^+^ MSC improved homing to recipient tissue	25
C57BL/6 or BALB/b TCD BM cells	BALB/c or C57BL6/-Ly-5.1 mice	5-6 Gy	Syngeneic, allogeneic, or 3rd party BM	0.25 × 10^6^; days 0, 4, 7, 10, 14	Syngenic MSCs increase engraftment, donor MSCs increase rejection, 3rd party MSCs no effect	14
Human PB CD34^+^cells	NOD/SCID mice	3 Gy	Autologous (PB) or allogeneic (BM), human	1 × 10^6^	Increased donor CD45^+^ with either MSC source, allogeneic MSCs increased myeloid engraftment and megakaryocytopoiesis	15

*MSCs* indicates mesenchymal stromal cells, *HSC* hematopoietic stem cell, *UCB* umbilical cord blood, *NOD*/*SCID* nonobese diabetic/severe combined immunodeficiency, Gy gray, *BM* bone marrow, *NR* not reported, *TCD* T cell depleted, *PB* peripheral blood, *PA-MSC* plastic-adherent MSC, *NK* natural killer

Different subsets of MSCs have been evaluated to potentiate engraftment in murine models. The infusion of CD271^+^ MSCs, a potential marker of precursor MSCs, isolated from human BM has been compared to the infusion of standard MSC (PA-MSC) for engraftment of human CD133^+^ peripheral blood (PB) cells into NOD/SCID mice (Table 1) [24]. In this study, CD271^+^ MSCs contained the entire colony-forming unit (CFU)-fibroblast activity (with none in CD271^−^ fraction) and had 1–3 fold higher proliferation compared to standard MSCs (so-called plastic adherent or PA-MSCs) [24]. While both MSC subsets increased engraftment, CD271^+^ MSCs resulted in greater CD45^+^ donor cell engraftment than PA-MSC [24]. STRO-1, which is expressed by adult BM fibroblast colony-forming units, has been thought to define a precursor subset of MSCs [25]. Similar to studies of CD271, both STRO-1^+^ and STRO-1^−^ MSCs have been shown to increase BM, PB and spleen engraftment of human CB cells in this model, although STRO-1^−^ MSCs are superior in this effect while STRO-1^+^ MSCs have greater migratory potential [25].

MSC donor source and dose may also impact the effect of MSC on engraftment. In two separate murine models (multiple minor mismatched and MHC mismatched), syngeneic BM MSCs significantly increase engraftment, allogeneic BM MSCs significantly decrease engraftment, and third-party BM MSCs have no effect [14]. In contrast, in a NOD/SCID model autologous or allogeneic MSCs comparably increase BM myeloid engraftment of human CD45^+^ cells [15]. This suggests that at least in a murine model of HCT, MSCs are not immunoprivileged, and that the donor source of MSCs can lead to profound differences in engraftment depending on the transplant model. Further, MSCs appear to have a greater impact on engraftment at lower HSC doses. Fetal lung MSCs increase the percent of human CD45^+^ cells engrafting in the BM by 3–4 fold, which was most pronounced at lower CD34^+^ cell doses (0.03-0.1 × 10^6^) [21]. Studies utilizing allogeneic MSCs found that promotion of myeloid engraftment was greatest with HSC doses <1 × 10^6^ giving further indication for a threshold level of the MSC effect on engraftment [15].

The capacity of MSCs to enhance engraftment has also been studied in large animal models (Table 2). In an in-utero human-sheep xenograft model, co-transplantation of human BM MSCs increased PB and BM engraftment of human CD34^+^ BM cells [16]. Donor source did not impact the ability of MSCs to promote graft acceptance, as both allogeneic and autologous MSCs had similar effects [16]. MSCs had no effect in two canine models of HCT using different MSC and HSC donor sources [26, 27]. Third-party MSCs (either from primary culture or an immortalized clonal population) had no impact on the engraftment of canine haploidentical BM cells following total body irradiation (TBI) when compared to BM cells alone, with half of animals rejecting grafts and half developing fatal acute GVHD [26]. Three dosing schedules were studied, but the sample size in each was too small to make inferences about dosing [26]. The authors hypothesize that post-transplant immunosuppression may be required in this model in order to see an impact of MSCs [26]. Donor-derived BM MSCs also had no impact on engraftment in a canine dog leukocyte antigen-identical transplant model using non-myeloablative conditioning (1 Gray), a model in which co-stimulation (CTLA4) blockade or anti-CD154 has been successful [27]. Failure of MSCs in these canine models may be attributed to numerous reasons, including source of MSCs, intensity of conditioning, absence of post-transplant immunosuppression, and/or the dosing schedule of MSCs. Finally, MSCs have been studied in a nonhuman primate model of autologous CD34^+^ intra-BM transplant, wherein autologous BM MSCs increased the percent of donor CFUs found in recipient BM, although the primates were not followed long term [28].

**Table 2:** Use of MSCs to promote engraftment in large animal models

HSC source	Model	Conditioning; Immune suppression	MSC source	MSC dose	Outcome	Ref
Human CD34^+^BM cells	Fetal sheep xenograft	None; n/a	Human autologous & allogeneic BM	5 × 10^4^ - 7.5 × 10^5^	Increased engraftment in PB and BM, no difference between autologous & allogeneic	16
Haploidentical BM cells	Canine	9.2 Gy; n/a	Allogeneic BM (3rd party), immortalized clonal populations or primary MSC culture	3 dosing schedules: 30 × 10^6^/kg 3×/wk ×1 wk, then 2×/wk; 15 × 10^6^/kg 5×/wk; 1 × 10^6^/kg 3×/wk	No effect; 50 % graft rejection & 50 % fatal acute GVHD	26
DLA-identical BM cells	Canine	1 Gy; MMF and CSA	Donor-derived BM	1.2 - 1.8 × 10^6^/kg day 0, 1.1 - 1.3 × 10^6^/kg day 35	No effect, uniform graft rejection at median of 8 weeks after initial donor engraftment	27
Autologous CD34^+^ BM cells (intra-BM)	Nonhuman primate	5.5 Gy ×2 doses or Bu; n/a	Autologous BM	NR	1.6-6 fold increase in donor CFUs in BM	28

*MSCs* indicates mesechymal stromal cells, *HSC* hematopoietic stem cells, *BM* bone marrow, *PB* peripheral blood, *Gy* gray, *Bu* busulfan, *NR* not reported, *CFUs* colony-forming units, *wk* week, *GVHD* graft-versus-host disease, *DLA* dog leukocyte antigen, *MMF* mycophenolate mofetil, *CSA* cyclosporine

#### Clinical Trials of MSCs to Promote Engraftment

As summarized in Table 3, a number of clinical trials have been published demonstrating the use of MSCs to promote HSC engraftment. These trials have all utilized a variety of MSC and HSC donor sources and a variety of MSC culture conditions making it difficult to directly compare studies. They are exclusively phase I and II trials that provide evidence of safety and supportive evidence for future larger phase III trials. These studies largely use allogeneic or MHC unmatched random donor third-party MSCs in an allogeneic HCT setting, although one of the earliest studies utilized autologous MSCs to support engraftment of autologous PB HSCs following high dose chemotherapy for breast cancer [29]. Although this study found no difference in neutrophil and platelet engraftment compared to a historical control group, it is the only study to use autologous MSCs and the only following autologous HCT [29]. Two patients initially enrolled on study did not receive their autologous MSCs due to persistence of BM involvement by breast cancer, highlighting a potential concern of using autologous MSCs in the malignant disease setting [29].

**Table 3:** Clinical trials of MSCs to promote engraftment

Patient population	HSC source	Conditioning; Immune suppression	MSC source	MSC dose	MSC culture conditions	Outcome	Ref
Adult breast cancer, *n* = 28	Autologous PB	Cy/Thio/Carbo; n/a	Autologous BM	Day 0 − +1: ≥ 1 × 10^6^/kg	FBS; Fresh or cryopreserved (*n* = 8); Passages NR	Engraftment of neutrophils 8 d & platelets 8.5 d; No difference compared to historical control group	29
Pediatric HR acute leukemia, *n* = 8	UCB	Cy/TBI or Bu/Mel (age <1 yr) & eATG/MP; CSA & MP	Haplo BM (parent)	Day 0: 2.1 × 10^6^/kg; Day 21: 1 × 10^6^/kg, *n* = 3	PlasmaLyte A, 5 % HSA; Cryopreserved; P1-P4	100 % donor chimerism (d 21), engraftment of neutrophils 19 d & platelets 53 d; 14 % gr II-IV aGVHD, no cGVHD; No difference compared to historical control group	30
Pediatric leukemia or HLH, *n* = 13	UCB	TBI or chemotherapy-based; CSA +/− steroids	Haplo BM (parent)	Day 0: 1.9 × 10^6^/kg	FBS; Fresh or cryopreserved; P2-P3	85 % donor engraftment, engraftment of neutrophils 30 d & platelets 32 d; 31 % gr II-IV aGVHD (0 % gr III-IV), 0 % cGVHD; ↓ gr III-IV aGVHD (*p* = 0.05), otherwise no difference compared to historical control group	31
Pediatric hemoglobinopathy, SCD (*n* = 4) or thalassemia major (*n* = 2)	UCB (1 single, 3 double) or UD BM (2)	Flu/Mel/alemtuzumab; CSA & MMF	Haplo (1) or third-party (5) BM	Day 0: 2 × 10^6^/kg; Day 2: 2 × 10^6^/kg	FBS; Cryopreserved; Passages NR	2 with primary graft failure & 2 with secondary graft failure, 4 deaths; Study prematurely terminated	32
Adult HR hematologic neoplasms, *n* = 9	UCB + third-party TCD PB HSC	Myeloablative; CSA & MP	Third-party BM (same as HSC; *n* = 7 haplo)	Day 0: 1.2 × 10^6^/kg	FBS; Cryopreserved; P1-P3	100 % donor engraftment (51 d), engraftment of neutrophils 12 d & platelets 44 d; 44 % gr II-IV aGVHD (0 % gr III-IV), 13 % cGVHD; No difference compared to concurrent control group	33
Pediatric & adult patients, leukemia (*n* = 3) or NMD (*n* = 4), indication graft failure in 3	MSD BM (1) or PB (2); UD BM (1), PB (2), or UCB (1)	Myeloablative (3) or RI (4) +/− ATG (6); CSA +/− MTX (4)	Haplo (4) or MSD (3) BM	Day 0: 1 × 10^6^/kg	FBS; Fresh vs cryopreserved NR; P2-P3	100 % donor engraftment, engraftment of neutrophils 12 d & platelets 12 d; 29 % gr II-IV aGVHD (0 % gr III-IV), 14 % cGVHD; No comparison group	34
Adult hematologic malignancy, *n* = 46	MSD BM or PB	Myeloablative; CSA & MTX	MSD BM	Day 0: 1, 2.5, or 5 × 10^6^/kg (actual dose)	FBS; Cryopreserved; Passages NR	Engraftment of neutrophils 14 d & platelets 20 d; 28 % gr II-IV aGVHD, 61 % cGVHD; No comparison group	35
Adult hematologic neoplasm, poor hematological recovery post-HCT, *n* = 6	Haplo TCD or MSD PB	TBI or chemotherapy-based +/− ATG (haplo); CSA +/− MTX (MSD)	Haplo or MSD BM (same as HSC)	1 × 10^6^/kg, day +159.5 (median)	Ultroser G serum substitute; Fresh vs cryopreserved NR; P2-P3	No response in 4 patients; 2 patients in CR1 with prompt neutrophil recovery (d 5 & 15) and platelet recovery (d 12 & 21); No comparison group	36
Pediatric patients, Hematologic malignancy (*n* = 11) or NMD (*n* = 3)	Haplo TCD PB CD34^+^	TBI or chemotherapy-based (64 %); NR	Haplo BM (same as HSC)	Day 0: 1.6 ×10^6^/kg (mean)	FBS; Fresh or cryopreserved; P1-P3	100 % donor engraftment, engraftment of neutrophils 12 d & platelets 10 d (mean); 38 % gr II-IV aGVHD (0 % gr III-IV), 7 % cGVHD; Faster reticulocyte (*p* = 0.03) & leukocyte recovery (*p* = 0.009), otherwise no difference compared to historical control group	37
Adult & young adult patients, hematologic malignancy, *n* = 30	MSD PB or BM	Cy/TBI or Bu/Cy; CSA & MTX; open-label randomization to control or MSC group, *n* = 15 per group	MSD BM	Day 0: 3.3 × 10^5^/kg	FBS; Fresh vs cryopreserved NR; Passages NR	5 patients in MSC group did not receive MSCs due to failed expansion; No difference in time to neutrophil & platelet engraftment; decreased incidence aGVHD (11.1 vs 53.3 %) & cGVHD (14.3 vs 28.6 %); comparable rates of infection; increased relapse (60 vs 20 %) and decreased DFS (30 vs 66.7 %) and OS (40 vs 66.7 %), no p-values reported	38

*MSCs* indicates mesenchymal stromal cells, *HSC* hematopoietic stem cell, *PB* peripheral blood,* Cy* cytoxan, *Thio* thiotepa, *Carbo* carboplatin, *n*/*a* not applicable, *BM* bone marrow, *FBS* fetal bovine serum, *NR* not reported, *d* days, *MSD* matched sibling donor, *CSA* cyclosporine, *MTX* methotrexate, *aGVHD* acute graft-versus-host disease, *cGVHD* chronic graft-versus-host disease, *NMD* non-malignant disease, *haplo* haploidentical, *TCD* T cell depleted, *TBI* total body irradiation, *DFS* disease-free survival, *OS* overall survival, *HR* high-risk, *UCB* umbilical cord blood, *eATG* equine anti-thymocyte globulin, *MP* methylprednisone, *HSA* human serum albumin, *CR1* complete remission 1, *HLH* hemophagocytic lymphohistiocytosis, *SCD* sickle cell disease, *UD* unrelated donor, *Flu* fludarabine, *Mel* melphalan

Several clinical trials have evaluated the use of peri-transplant MSCs following CBT using third-party or haploidentical MSCs (Table 3). Following infusion of haploidentical MSCs, pediatric patients with high-risk acute leukemia had engraftment and rates of GVHD comparable to historical controls; all 8 patients engrafted with a low rate of aGVHD [30]. A similar study utilized haploidentical MSCs in pediatric patients with hematologic malignancies and hemophagocytic lymphohistiocytosis [31]. Compared to a historical control group, there were comparable rates of engraftment, although patients receiving MSCs had significantly less grade III-IV aGVHD [31]. Statistically more patients in the historical control group received granulocyte-colony stimulating factor compared to patients in the MSC group, which may have masked a difference in neutrophil engraftment [31]. The majority of study patients also received more than the minimum CD34^+^ cell dose, which the authors suggest may have impacted the ability to demonstrate the effect of MSCs in potentiating engraftment [31]. Interestingly, no patients in either of these studies developed cGVHD [30, 31]. A third study in pediatric patients with hemoglobinopathies utilized MSCs from third-party or haploidentical donors to potentiate CB engraftment; two patients died from transplant-related complications prior to achieving engraftment and the remaining two patients had graft rejection with auto-reconstitution [32]. This study was halted prematurely and highlights the significant engraftment barrier in patients with hemoglobinopathies following CBT [32]. Finally, a study of adult patients with high-risk hematologic malignancies found comparable rates of engraftment in patients receiving donor-derived (mostly haploidentical) MSCs to that of a concurrent control group [33]. Both groups of patients received CB concurrent with third-party TCD PB HSCs [33]. All patients receiving MSCs had rapid donor engraftment with a low incidence of cGVHD and no grade III-IV aGVHD [33].

MSCs have been utilized in the transplant of BM or PB HSCs from sibling, unrelated, and haploidentical donors (Table 3). The majority of these studies occurred in relatively homogenous populations of patients and used homogenous MSC and HSC sources; Le Blanc et al. [34], for example, infused haploidentical or matched-sibling donor (MSD) MSCs into a heterogeneous patient population making it difficult to garner much from this trial aside from safety. This trial included pediatric and adult patients with leukemia or NMD, conditioned patients with myeloablative or reduced-intensity regimens, and utilized HSCs from MSD BM or PB, MUD BM or PB, or CB [34]. Two studies evaluated MSCs in adult patients with hematologic malignancies, although the lack of even a historical comparison group makes both studies difficult to interpret [35, 36]. MSD BM or PB engraftment was rapid in patients who received peri-transplant MSCs from their MSD [35]. The second study employed MSCs in the setting of poor hematological recovery (full donor chimerism with <10 % cellularity in BM) [36]. The six patients received donor-derived MSCs a median of 160 days post haploidentical or MSD HCT, with two patients having prompt neutrophil and platelet engraftment [36]. An additional study infused haploidentical MSCs into pediatric patients with primarily hematologic malignancies to potentiate haploidentical HSC engraftment [37]. Patients receiving MSCs had significantly faster leukocyte and reticulocyte recovery when compared to a historical control group [37]. Although there were no significant differences in rates of GVHD, it is notable that there was no grade III-IV aGVHD in the MSC group [37].

In the only randomized trial of MSCs given peri-HCT to date, primarily adult patients with hematologic malignancies undergoing MSD HCT were randomized to standard GVHD prophylaxis (cyclosporine and methotrexate) or standard GVHD prophylaxis plus MSCs from their MSD (Table 3) [38]. There were significant difficulties in expanding MSCs; five patients randomized to receive MSCs did not receive any and the median dose of MSCs was low at 3.3 × 10^5^/kg [38]. The authors report significantly decreased rates of acute and chronic GVHD in the MSC group and significantly increased rates of relapse, leading to decreased event-free survival and overall survival, although no statistical analyses are reported [38]. Data analysis was also not performed using an intent-to-treat analysis, therefore the effect of randomization was not preserved and significant differences between the two groups may have occurred leading to confounding of results.

In addition to being given early in HCT to support HSC engraftment, MSCs have been employed to promote ex vivo expansion of HSCs. In an elegant study by de Lima et al. [39], adult patients with hematologic malignancies receiving double CBT had the smaller CB expanded in ex-vivo co-culture with MSCs then infused on day 0 following receipt of the unmanipulated larger CB unit. Patients had significantly more rapid neutrophil and platelet engraftment and higher cumulative incidence of neutrophil and platelet engraftment when compared to a matched historical control group [39]. The expanded cord predominated early post-HCT, while the unmanipulated cord predominated long-term (>1 year). The authors attribute this observation to co-culture increasing progenitor cells committed to megakaryocyte and myeloid lineages and depleting cells important in long-term repopulation [39].

### Safety of MSCs in HCT

Numerous studies in a variety of conditions have thus far documented safety following infusion of MSCs, and a recent meta-analysis of 36 studies (including 11 HCT studies) demonstrated no association with acute infusion toxicity, organ toxicity, malignancy, infection or death with a significant association only with transient fever [40]. Based upon the immunosuppressive properties of MSCs, downstream effects on malignant cells and infections have been of particular concern in HCT patients. The previously described study by Ning et al. [38] is the only published study reporting an increase in relapse rate following MSC infusion, although as discussed, the study did not report statistical analyses and did not preserve the randomization. The inverse relationship found between GVHD and relapse in this study, however, does fit with numerous other studies documenting the same effect (likely due to decreased graft-versus-leukemia effect, GVL) and warrants continued long-term monitoring in patients receiving MSCs in the malignant setting.

Increased rates of infectious complications have been reported in several small non-randomized trials, but as previously stated no association was found in a recent meta-analysis [40]. CMV viral loads and the rate of CMV disease were higher than expected in thirty-one patients receiving MSCs as treatment for GVHD or hemorrhagic cystitis. All cases of CMV disease, however, occurred in a GVHD-affected organ and most were described as mild [41]. Further, this study was single arm without even a historical comparison group reported, so it is difficult to ascribe causative effect. A cohort of pediatric patients receiving MSCs as treatment for steroid-refractory GVHD had comparable rates of CMV, EBV and adenovirus when compared to historical controls, although adenoviral infection resulted in decreased survival, particularly when it occurred following MSC infusion [42]. While an effect on adenovirus-specific T cells was found in vitro, no in vivo effect was found, and the authors note significantly higher rates of HLA mismatched grafts in the MSC group [42]. Finally, a recent retrospective analysis of 1021 patients transplanted at the Karolinska University Hospital found MSC infusion to be a significant predictor of post-transplant lymphoproliferative disease on multivariate analysis, a finding that has not been previously reported [43]. As none of these studies were randomized trials, they serve as further reminders that long-term monitoring in patients receiving MSCs is necessary, particularly in future randomized trials.

## Conclusions

### Future Directions for MSCs in HCT Setting

As shown in Table 3, clinical trials of MSCs have been very heterogeneous in terms of HSC source, MSC source, and MSC culture conditions. Although MSCs have been described as immunoprivileged, pre-clinical studies suggest that MSC donor source and culture conditions alter their immunoregulatory potential. MSCs upregulate MHC class I and express MHC class II under inflammatory conditions, and MHC-mismatched murine MSCs undergo specific immune-mediated rejection [44]. In a non-myeloablative experimental setting, donor MSCs stimulate immune-mediated graft rejection whereas host MSCs promote engraftment [14]. Third-party MSCs, which have been the largest source in clinical trials, showed no response, which the authors hypothesize was secondary to their rejection [14]. Pre-clinical studies reveal that MSC donor source may not be as important in severely immunocompromised recipients, including NOD/SCID mice, fetal sheep, or in the setting of significant conditioning (e.g. TBI) [13, 15, 16, 21, 23–25, 45]. These data indicate that donor source of MSCs may be most critical in the setting of sublethal or minimal conditioning, such as that often used in the NMD setting. Culturing in FBS may also enhance MSC immunogenicity [46]. The majority of clinical trials have also used cryopreserved, serially expanded MSCs. Freshly thawed MSCs have impaired in vitro T cell suppression secondary to a reversible heat shock response with diminished IFN-γ-dependent IDO up-regulation [47]. Serial expansion of MSCs results in clonal impoverishment, telomere shortening and increased cell senescence, and MSC passage correlates with response and survival from acute GVHD suggesting a relationship with functional impairment [41, 46].

The majority of pre-clinical studies have demonstrated that MSCs can potentiate HSC engraftment, while clinical trials have been difficult to interpret broadly due to being small, largely non-randomized trials. These studies have furthered evidence of safety in HCT populations and give suggestion for potential impact of MSCs on engraftment, particularly following haploidentical transplant [37]. Differences in clinical efficacy may relate to the mechanisms of graft failure in different donor settings- HSC homing or cell number is likely more important in CBT, whereas graft failure following haploidentical transplant is predominantly immune-mediated [48]. This suggests that MSCs could be particularly efficacious in promoting engraftment of haploidentical HSCs. Pre-clinical studies also suggest that MSCs have a greater impact on engraftment at lower HSC doses, therefore may have greater impact in this clinical setting [15, 21]. Clinical trials to date also support future studies of MSCs to prevent GVHD, with a significant decrease in aGVHD reported in one study and no aGVHD reported in two additional studies [31, 33, 37]. MSCs may have greater potential to impact on GVHD when the inflammatory state is lower. The use of MSCs to prevent GVHD may therefore be more successful than its use to treat active GVHD. Finally, caution is indicated when MSCs are given in a malignant disease setting, both due to the potential for increased rates of relapse [38] and to the known inverse ratio between relapse and GVHD/GVL, suggesting that MSCs may be more safe and more useful in the NMD setting. Regardless of the specific clinical setting chosen, sufficient evidence of safety and early evidence of efficacy exists regarding MSCs to facilitate HSC engraftment and to prevent GVHD, warranting the completion of larger, more homogenous, and ultimately, randomized clinical trials. Additional pre-clinical studies of MSCs, particularly in more immunocompetent rodents and in large animal models, are still warranted to further establish the ideal source of MSCs (and HSCs) and MSC mechanism of action, particularly in potentiating engraftment.
